# Brain Arousal as Measured by EEG-Assessment Differs Between Children and Adolescents With Attention-Deficit/Hyperactivity Disorder (ADHD) and Depression

**DOI:** 10.3389/fpsyt.2021.633880

**Published:** 2021-10-28

**Authors:** Christoph Berger, Alexander Dück, Felicitas Perin, Katharina Wunsch, Johannes Buchmann, Michael Kölch, Olaf Reis, Ivo Marx

**Affiliations:** Department of Psychiatry, Neurology, Psychosomatics, and Psychotherapy in Childhood and Adolescence, Rostock University Medical Center, Rostock, Germany

**Keywords:** EEG, vigilance, brain arousal, ADHD, depression, children, vigall

## Abstract

**Objective:** Disturbed regulation of vigilance in the wake state seems to play a key role in the development of mental disorders. It is assumed that hyperactivity in adult ADHD is an attempt to increase a general low vigilance level via external stimulation in order to avoid drowsiness. For depression, the avoidance of stimulation is interpreted as a reaction to a tonic increased vigilance state. Although ADHD is assumed to start during childhood, this vigilance model has been barely tested with children diagnosed for ADHD so far.

**Methods:** Resting-state EEG (8 min) measures from two groups of children diagnosed with either ADHD [*N* = 76 (16 female, 60 male), age: (mean/SD) 118/33 months] or depression [*N* = 94 (73 female, 21 male), age: 184/23 months] were analyzed. Using the VIGALL toolbox, EEG patterns of vigilance level, and regulation were derived and compared between both groups. In correlation analysis, the relations between vigilance measures, attentional test performance (alertness and inhibition), and mental health symptoms were analyzed.

**Results:** Children with ADHD differed from children with most prominent depressive symptoms in brain arousal regulation and level, but EEG vigilance was not related to behavior problems and not related to the attentional test performance. Brain arousal was dependent on the age of the participant in the whole sample; younger children showed lower vigilance stages than teenagers; this effect was not present when analyzed separately for each diagnostic group. EEG assessment time and received medication had no effect on the EEG vigilance.

**Discussion:** Although based on a small sample, this explorative research revealed that EEG vigilance level is different between children with ADHD and with depression. Moreover, even the standard procedure of the clinical routine EEG (resting state) can be used to differentiate brain arousal states between participants with ADHD and depression. Because routine EEG is not specialized to vigilance assessment, it may not be sufficiently sensitive to find vigilance–symptomatology associations. Further research should address developmental changes in EEG measurements in children and use bigger samples of participants within the same age range.

## Introduction

The arousal regulation model of affective disorders (1) keeps the focus on a dysfunctional brain arousal state in wakefulness regarding its level and maladaptive autoregulation and assumes a causal connection of brain arousal to psychiatric disorders of the affective system like depression and mania. In terms of dysfunctional brain arousal state, the brain can be up- or downregulated. Adult patients with depression often show upregulated brain states, resulting in inner tension and inhibition of drive (2) and prolonged sleep latency (3). The opposite, downregulated brain states with short sleep latency and a high prevalence of excessive daytime sleepiness and sleep and circadian disorders are related to ADHD (4) and mania (1). According to that model, many behavioral patterns have the autoregulatory function to compensate for dysfunctional instability of brain arousal (e.g., stimulus avoidance of individuals with depression in order to lower the inner tension and hyperactivity in individuals with ADHD to avoid drowsiness) and these patterns can result not only in personal traits but also in clinically relevant behavioral syndromes, when vulnerable subjects are affected (1, 5).

Brain arousal regulation is mainly driven by coupled activity of the thalamus and the formatio reticularis (FR) in the brainstem. An activation of the medial part of the FR, described as the ascending reticular activation system (6), leads to an increased vigilance state and a desynchronized EEG. Projections from the activated FR to the thalamus lead to increased brain arousal, partly due to decreased inhibition of thalamic relay cells forwarding specific (e.g., sensoric) projections and partly due to non-specific thalamic projection systems, facilitating cortical activity (7). Other projections modulating brain arousal also exist from hypothalamus, limbic system, and the basal forebrain (8).

Like EEG-based assessment of different vigilance stages during sleep, it is also possible to identify different vigilance levels during wakefulness, from high alertness down to the onset of sleep. This vigilance shift before sleep is related to typical changes of brain potentials: A desynchronized non-alpha EEG without eye movements during high alertness is shifting to dominant alpha activity during relaxed wakefulness; alpha activity is then dissolving again with slow eye movements (SEM) occurring during drowsiness. This stage transits furthermore to dominant theta/delta activity with occurring patterns of transitions to sleep like vertex waves and finally with markers of sleep onset present, like sleep spindles, and K-complexes.

For a classification of these vigilance levels, the software tool VIGALL was developed by the Department of Psychiatry of the University of Leipzig, Germany (http://research.uni-leipzig.de/vigall/). VIGALL was used to validate the arousal regulation model in many studies on adult samples. For participants with diagnosis of depression, a hyperstable vigilance regulation was confirmed: More arousal and later decline were present in depressive patients compared to healthy controls (9). A clustering analysis revealed a more stable vigilance regulation pattern in individuals with diagnosis of depressive compared to controls (10). Higher arousal level and slower decline were related to more severe depressive symptoms (11). Interestingly, vigilance level of responders to antidepressant medications at baseline and also the decline after the therapy were both higher than those of non-responders (12). Similar effects on the vigilance level were shown by the same research group for individuals with depression after sleep deprivation (13).

An unstable arousal regulation may be present not only for affective disorders but also as the basic brain dysfunction for ADHD (14). Confirmed by a study report using VIGALL, individuals with diagnosis of ADHD had lower mean vigilance stages and a faster vigilance decline than healthy controls and furthermore arousal regulation predicted the retrospectively-assessed severity of childhood ADHD symptoms (15).

Because the VIGALL algorithm depends on a stable alpha rhythm in order to work properly, EEG measures from children <10 years should be analyzed with special caution. At the age of 7 years, a mean alpha peak frequency (APF) at 9 Hz will be reached and an APF of 10 Hz at the age of 15 years (16). Beside Alpha, Theta, and Delta band EEG activity is also relevant for the vigilance classification by the algorithm and can be different from adult EEG. About 25% of typically developing children and early adolescence show Theta and Delta slowing (17). Single arrhythmic pattern of Delta EEG activity in occipital and posterior–temporal regions are known as the “posterior slow waves of youth” and have its maximum expression between 8 and 14 years (16). Posterior rhythmic activity in the 2.5–4.5 Hz band occur with closed eyes at the age of 5–7 years and disappear up to the age of 15 years (16). Probably because of these well-known age-related EEG patterns, we found only one study report using VIGALL in a sample of children. In a study by Sander et al., resting-state EEG segments with closed eyes of 2 min length of children with diagnosis of ADHD were compared to age-matched healthy controls in the overall age range from 6 to 18 years. The authors confirmed model assumption of hyperstable arousal regulation of ADHD and found that the children with ADHD spend less time in the high aroused state and shift more often between the vigilance states (18). Additionally there seems to be evidence for a higher theta/beta ratio present in ADHD compared to healthy controls. This was confirmed in some studies but also one study revealed no differences between ADHD and the healthy control group, as described in a review of quantitative EEG as a possible biomarker in child psychiatry (19).

A different study comparing children with ADHD to healthy controls and using resting-state EEG together with behavioral and cognitive characteristics in a latent class analysis revealed a heterogeneity of EEG subgroups over all subjects, suggesting that there is no single resting-state profile dominant for children with or without ADHD (20).

To our knowledge, no study report exists about the evaluation of EEG vigilance using VIGALL in children and adolescents with depression. When looking for study reports using other physiological parameters related to the model of hyperarousal regulation in children with depression, the findings are mixed. The ultradian synchronization of sleep EEG rhythms was lower in children with major depression compared to healthy controls (21).This was associated with dampened amplitude of the circadian rest–activity cycle in that sample (22). Other studies showed mixed findings on objective sleep parameters (23) and there was also no clear direction whether cortisol level differences in children with depression exist compared to healthy controls (24, 25). One study reported changes in cardiac activity associated with depression, resulting in increased heart rate but no differences in heart rate variability (HRV) occurred (26).

Taken together, there are a limited number of studies in the literature about brain arousal regulation in children with psychiatric disorders compared to adults. Furthermore, it is not currently possible to claim that a particular brain arousal regulation type is predominantly associated either with externalizing behavior like ADHD or internalizing symptoms like depression in childhood. The same heterogeneous EEG profile distribution has been found for ADHD children and healthy controls (20). HRV and cortisol levels as markers of self-regulation did not differ in depressive compared to healthy children. Therefore, the present explorative study using vigilance EEG measures had the aim to examine whether the assumption of divergent arousal regulation types between ADHD and depression in adults can also be supported for children.

## Materials and Methods

### Participants

In this study, we examined two groups of children and adolescents aged 6–18 years, one group consisting of participants with a clinical diagnosis of depression or emotional disorders with most prominent depressive symptoms (ICD-10 codes: F32.x, F33.x, F43.2, F93.8), and one group of participants with a clinical diagnosis of ADHD (ICD-10 codes: F90.0; F90.1). The children and adolescents were inpatients and outpatients who were consecutively admitted to the Department of Child and Adolescent Psychiatry, Neurology, Psychosomatics, and Psychotherapy of the University Medicine Rostock in the years between 2015 and 2017. Clinical diagnoses were established by a team of experienced child and adolescent psychiatrists and psychologists according to the ICD-10 criteria. The diagnostic procedure included self- and other-informant psychiatric screening questionnaires [i.e., Child Behavior Check List /6-18R (CBCL), Teacher Report Form (TRF), and Youth Self-Report (YSR)], disorder-specific questionnaires [e.g., Beck Depression Inventory (BDI-II), Hamilton rating scale for depression (HAM-D), and Depression inventory for children and youth (DIKJ) in the depression group; Diagnostic system for psychiatric disorders according to ICD-10 and DSM-5 for childhood and adolescence (DISYPS-III): external rating report for ADHD (FBB-ADHS) in the ADHD group], intelligence quotient (IQ) testing [e.g., Hamburg-Wechsler-Intelligence-test for children (HAWIK-IV), Wechsler adult intelligence scale (WAIS-IV), Kaufman assessment battery for children (K-ABC-II), Wechsler Preschool and Primary Scale of Intelligence (WPPSI-III), and Culture fair intelligence test (CFT 20-R)], behavioral observations, as well as neuropsychological investigation of attentional functions in the ADHD group. The following exclusion criteria were defined: intellectual disabilities (F70-F79), neurological (e.g., seizure history), or severe endocrine (e.g., thyroid dysfunction) disorders known to affect brain function, head injury with loss of consciousness, lifetime schizophrenia spectrum disorder, autism spectrum disorders, a diagnosis of depression in the ADHD group, and vice versa.

**Figure 1:**
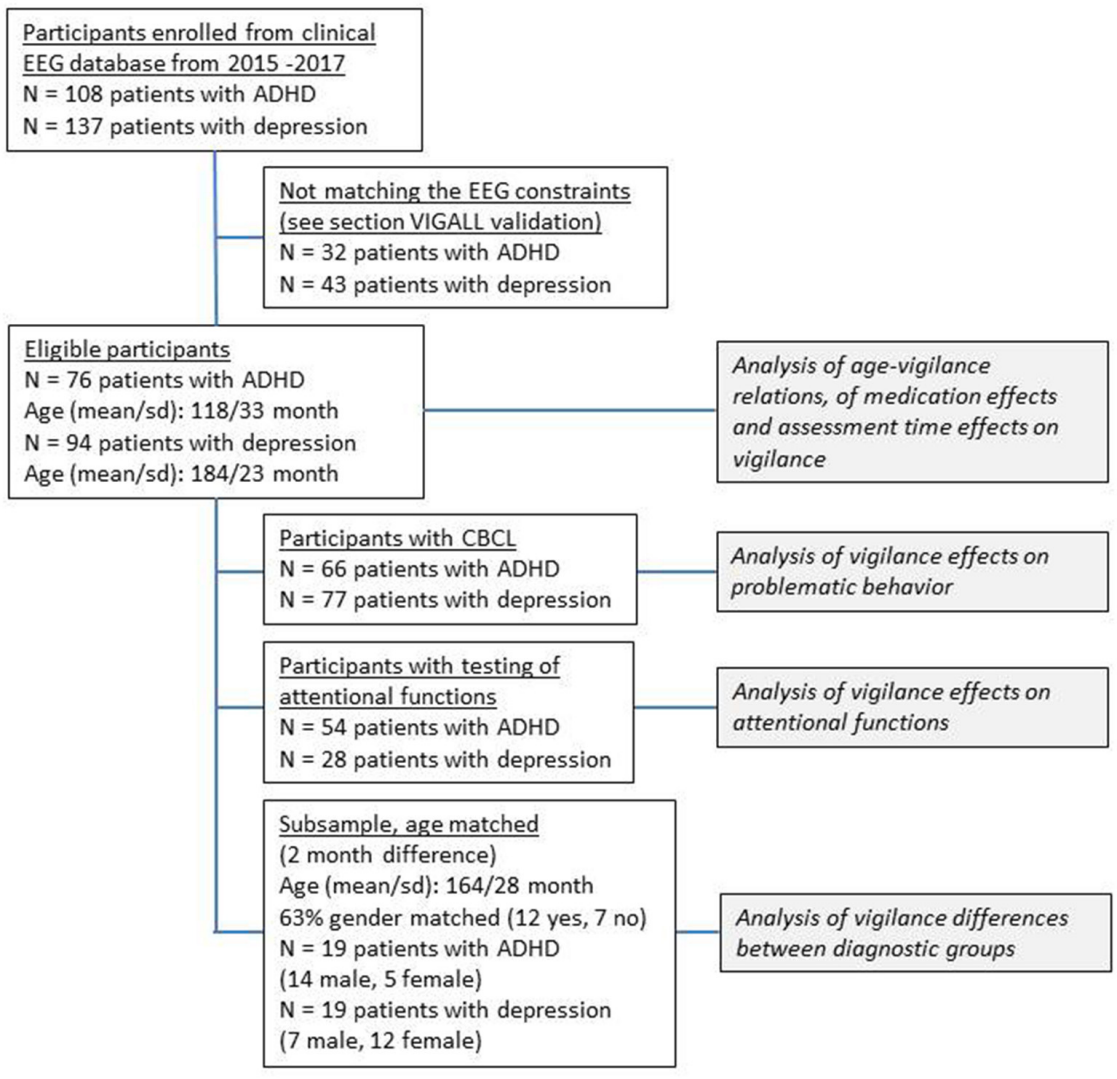
Study design flow chart. This is a sketch of the study design; in order to maximize the number of participants, we analyzed the effect of the covariates age, medication, and time of assessment on the EEG vigilance in the first subsample of all eligible participants.

The EEG data were originally acquired for clinical routine diagnostic purposes and retrospectively analyzed for this study. First, we identified patients with a diagnosis of depression or with emotional problems with most prominent symptoms of depression and patients with diagnosis of ADHD in the EEG database. From those patients, we excluded all individuals where the EEG validation criteria were not met, resulting in a sample of 76 individuals (16 female, 60 male) with ADHD and 94 individuals (73 female, 21 male) with most prominent depressive symptoms. This was the basis for covariate analysis of age, medication, and assessment time effects. On separate subsamples, we tested vigilance differences between the diagnostic groups and effects of vigilance on children's problematic behavior and on attentional performance. The study design is represented in the flowchart in Figure 1. We performed case–control matching with random case selection using the FUZZY plugin v.2.0.1., implemented in in SPSS 27. We allowed 2-month age differences in the matched pairs of individuals resulting in a sample of 19 individuals for analysis of vigilance differences between the diagnostic groups. Gender was not an explicit matching criteria, in order to keep the sample size not too low. Nevertheless, 63% of the individuals in this subsample were also gender matched.

The sample of eligible EEG measurement was used for covariate analysis of medication effects on the EEG vigilance; 68.8% of the patients were drug-naive, while the others were examined on medication: methylphenidate: *n* = 23 (13.5%), antipsychotics: *n* = 16 (9.4%), and antidepressants: *n* = 10 (5.9%). Because the received medication could probably influence brain arousal, we analyzed possible medication effects on brain arousal as described later. Both experimental groups differed from each other with regard to age. As expected, they also differed in their clinical profile, i.e., subjects in the depression group yielded higher depression scores, whereas subjects in the ADHD group yielded higher inattention scores. Clinical characteristics are presented in **Table 2**.

### EEG Acquisition

The EEG acquisition was conducted by a certificated medical technical assistant using the XLTEK EEG system (eeg32u amplifier, Natus Europe GmbH, Planegg, Germany). The EEG acquisition was done as part of routine clinical diagnostics. Continuous EEG was recorded while the participants were seated comfortably with closed eyes on a semi-reclined armchair. Nineteen electrodes were placed according to basic international 10–20 system (Fp1, Fp2, F7, F3, Fz, F4, F8, T3, C3, Cz, C4, T4, T5, P3, Pz, P4, T6, O1, and O2) and were referenced to linked earlobes. Ground electrode was placed between Fz and Cz electrode and one electrode on each wrist to measure the electrocardiogram (ECG). The impedance across all electrodes were quite similar and below 10 kohm. The sampling frequency was 512 Hz. The EEG data were recorded for about 13 min, including 10 min of normal breathing and 3 min of hyperventilation. In order to obtain similar artifact-reduced EEG for all participants, 8 min of the normal breathing part was used for further processing.

### EEG Preprocessing Pipeline

Further processing was done with the BrainVision Analyzer (Mesmed GmbH, Gilching Germany). The EEG data were preprocessed according to the manual of the VIGALL toolbox (https://research.uni-leipzig.de/vigall/), including the following steps: Filtering with Butterworth zero phase filter (0.5–70 Hz, notch at 50 Hz), creating 1-s segments, rough artifact screening by visual inspection, execution of independent component analysis (ICA), and exclusion of ICA components reflecting continuous artifacts like blinks, eye movements and cardioballistic artifacts, and marking of remaining artifacts. EEG data were screened for sleep graphoelements (sleep-spindles, K-complexes), but none were identified, which is expectable because the assistant was permanently monitoring the EEG recording during acquisition and prevents the proband from directly falling asleep.

### EEG Vigilance Classification

The consecutive segments of 1-s length were classified into six different EEG vigilance stages: 0, A1, A2, A3, B, and B2/3 (C was not observed) from wakefulness to drowsiness by using the add-on VIGALL 2.1 for the BrainVision Analyzer. VIGALL uses source localization in different frequency bands with LORETA. Further information about VIGALL, which is licensed under GPL3, is available at https://github.com/danielboettger/VIGALL or at https://research.uni-leipzig.de/vigall/.

VIGALL uses continuous electro-occulogram (EOG) data to detect SEM for discrimination B1 stage from stage 0. Because EOG data were not recorded, we were using the particular ICA component reflecting SEM, which can be determined by its typical topography. Furthermore, we omitted the Delta range during classification, because of the absence of sleep pattern and neuronal Delta range activity and in order to suppress probably occurring non-neuronal artifacts in the 2–4 Hz range as described before (11).

In order to keep data comparable to prior reported EEG vigilance research (11), we used similar methods for arousal analysis. We calculated the arousal stability index, based on 1-min intervals (interval 1, segments 1–60; interval 2, segments 2–61; etc.; for scoring criteria, see Table 1). Furthermore, we calculated the mean arousal level from the whole EEG acquisition period and the percentage of EEG vigilance stage occurrence (number of segments of one stage ^*^ 100/number of all artifact-free segments).

**Table 1:** Scoring criteria of the arousal stability index.

**Scoring criteria**	**Score**
>2/3 of all segments classified as 0 or A1	8
≥2/3 of all segments classified as 0 or A1, A2, A3	7
≥1/3 of last 160 s classified as B1	6
≥1/3 of second 160 s classified as B1	5
≥1/3 of first 160 s classified as B1	4
≥1/3 of last 160 s classified as B2/3	3
≥1/3 of second 160 s classified as B2/3	2
≥1/3 of first 160 s classified as B2/3	1

*The arousal stability index quantifies the extent of the arousal regulation. This index was developed by the VIGALL research group (11); lower values mean earlier arousal decline*.

### VIGALL Validation

The VIGALL toolbox has also some limitations in application, in particular with respect to variant alpha rhythms. Therefore, we included only the data with the following constraints:

1. Alpha frequencies between 8.5 and 12.5 Hz.
2. The amount of artifacts was <15%.
3. A plausible automatic detection of alpha activity: the absolute power of automatic detected alpha activity was more than 25,000 and the alpha activity was detected on early segments.
4. Less than 95% of the segments are classified as 0 or B1, in order to exclude low-voltage EEG.

### Statistical Analyses

Statistical analyses were performed with SPSS Statistics 27 (IBM Corp; Armonk, NY, USA). This study has an explorative approach because it was conducted retrospectively on a convenience sample of in- and outpatients stratified for diagnosis, and therefore, the data incorporate some covariates that have to be considered in the analysis strategy. Firstly, we tested the possible effects of the following covariates on the vigilance level and regulation scores: time of EEG acquisition, medication, and age. The diagnostic groups of ADHD and depressive patients were different in age and gender; therefore, we additionally analyzed the relation of age on the EEG vigilance for each diagnostic group separately; 31.2% of the included eligible participants also received psychopharmaceutic medication at the time of EEG measurement, and we addressed possible effects of medication in separate statistical analysis as described further on. Nevertheless, all included participants had symptoms related to ADHD or depression and met the diagnostic criteria for one of these conditions despite having started psychotherapeutic treatment at the time of the EEG. Therefore, we assumed that the received medication did not compensate effects of ADHD or depression diagnosis on the EEG vigilance In order to examine this assumption, we tested the effects of medication for each diagnosis separately. We tested with the Mann–Whitney *U*-test differences in arousal (stability and mean vigilance) related to medication (yes/no). With the Kruskal–Wallis test, we additionally analyzed the effects of the different medication types in the depressive group (no/antipsychotics/antidepressants) and in the ADHD group (no/antipsychotics/stimulants). Because we did not find any effects of the received medication on the EEG vigilance and because we would not decrease the sample size in this explorative study, we performed the following analysis on the whole sample, regardless of medication.

Secondly, we tested for significant associations between EEG vigilance and diagnostic assessment of problematic behavior and of attentional performance: In a correlation analysis with Spearman's correlation, we tested whether problematic behavior in any of the eight sub scores of the CBCL (27) as well in the total score and the scores of externalizing and internalizing behavior is associated with a particular EEG arousal regulation or arousal level. The association of EEG vigilance with the attentional performance was analyzed with the Spearman's correlation of vigilance stability, mean vigilance, and occurrence with A1 and B2/3 vigilance level with amount of Go errors of the Go/NoGo task (standardized percent range) and with median of reaction times of the alertness task, corrected for age-related variability (tonic alertness). Both attentional performance measures were assessed by the computer-based Test Battery for Attentional Performance (28).

Thirdly, we tested for differences in arousal regulation and arousal level (mean EEG vigilance and amount of segments in A1 and B2/3) between children with ADHD and children with depression with Mann–Whitney *U*-test, because of the non-normality of the data. Because of the above-described age effects on the EEG in childhood, we performed all tests for group effects (ADHD vs. depression) on a subsample, parallelized for age and partly for gender.

Because this study has an explorative character, we did not apply a correction for multiple testing, but limited the correlation analysis of single vigilance classification levels only to the vigilance level A1 and B2/3, because about 81% of EEG acquisition time was classified as one of these levels (see Table 5).

Effect sizes were reported as Pearson's correlation coefficient *r* for Mann–Whitney *U*-test and as η^2^ for Kruskal–Wallis test, with *r* = Z/$\sqrt{n}$ and η^2^ = (*H* – *k* + 1)/(*n* – *k*) [*n* = number of subjects, *k* = number of groups, test variables *Z* (Mann–Whitney *U*-test), and *H* (Kruskal–Wallis test)].

We report how we determined our sample size, all data exclusions, all manipulations, and all measures in the study (29).

## Results

### Study Sample

Internalizing and externalizing and problematic behavior scores are listed in Table 2. As expected, the ADHD group showed more attentional problems as well as more rule breaking and aggressive behavior. This is also reflected by a higher score of externalizing behavior. In contrast, the depressive group showed more withdrawn- and anxious-depressive behavior and a higher internalizing score. Table 3 shows the median of tonic alertness reaction time from the alertness task and the Go errors from the Go/NoGo task. Patients with diagnosis of ADHD were making more errors in the Go task than, and similar reaction times to, the depressive patients, but both groups revealed task performance in a clinically normal range.

**Table 2:** Behavioral problems.

	**ADHD (*****N*** **=** **66)**		**Depression (*****N*** **=** **77)**		
	**Mean**	**SD**	**Mean**	**SD**	***Z*; *p*; *r***
Age in months	117.11	29.65	183.65	22.51	**−9.17;** **<0.001; 0.77**
Internalizing score	59.21	9.93	65.95	7.15	**3.91;** **<0.0001; 0.33**
Externalizing score	64.44	12.10	56.16	10.64	**4.26;** **<0.0001; 0.36**
Total score	64.60	9.51	62.84	7.26	1.81; 0.07; 0.15
Withdrawn/Depressed	59.46	9.59	64.41	7.93	**3.69;** **<0.001; 0.31**
Somatic complaints	58.63	7.59	60.92	8.91	1.63; 0.103;0.14
Anxious/Depressed	58.42	10.58	64.95	7.39	**4.26;** **<0.0001; 0.36**
Social problems	60.89	10.02	57.24	7.53	**2.30; 0.016; 0.19**
Thought problems	58.74	8.56	61.82	8.82	**1.79; 0.022; 0.15**
Attention problems	65.32	8.09	59.30	7.21	**4.38;** **<0.0001; 0.37**
Rule-breaking behavior	74.51	84.08	59.75	9.12	2.73; 0.006; 0.23
Aggressive behavior	65.89	11.57	57.07	9.15	**4.66;** **<0.0001; 0.39**

*The Mann–Whitney U-test was used for testing group effects. r, Pearson's correlation index as effect size r = Z/$\sqrt{n}$; n, number of all participants; SD, standard deviation. Shown are mean and SD of standardized T-values. The statistical significant group differences are shown in bold*.

**Table 3:** Attentional task performance.

	**ADHD (*****N*** **=** **44)**		**Depression (*****N*** **=** **26)**		
	**Mean**	**SD**	**Mean**	**SD**	***Z*; *p*; *r***
Age in months	121.98	30.81	178.07	29.19	**−5.69;** **<0.001; 0.68**
Tonical alertness, reaction time (ms)	290.22	53.0	288.22	61.24	−0.86; 0.853; 0.1
Error in go task (percent range)	33.21	11.34	42.27	17.31	−1.99; 0.046; 0.24

*The Mann–Whitney U-test was used for testing group effects. r, Pearson's correlation index as effect size r = Z/$\sqrt{n}$; n, number of all participants; SD, standard deviation. The statistical significant group differences are shown in bold*.

### Comparison of Vigilance Measures Between the Diagnostic Groups

**Figure 2:**
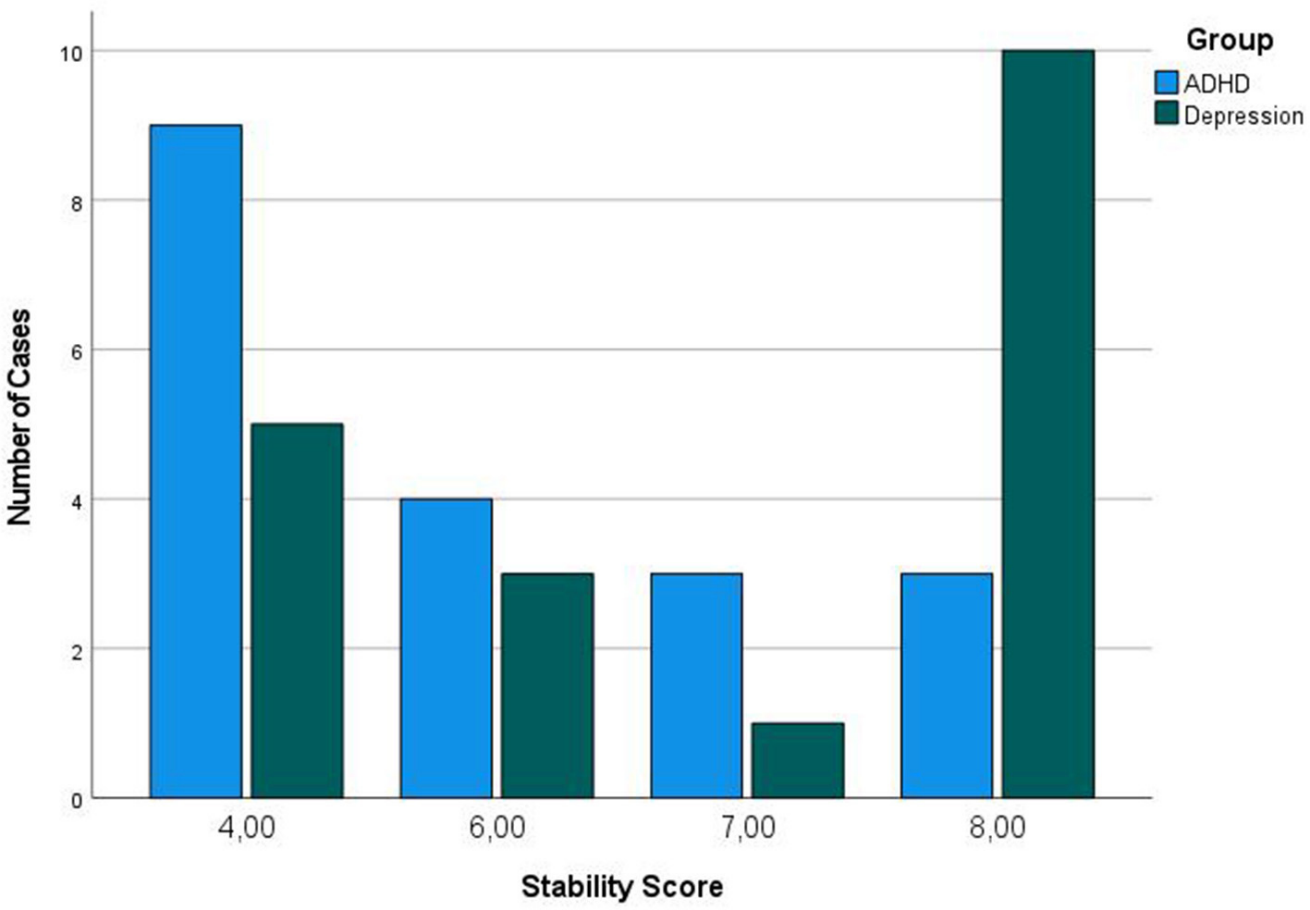
EEG stability distribution between diagnostic groups. Here are shown the vigilance stability index distribution in the age-matched subsample with 19 participants in each diagnostic group. For stability score, see Table 1; lower stability value means faster vigilance decline. The stability distribution was different between the groups: Pearson Chi-Quadrat = 10.9, *df* = 3, *p* = 0.012.

We tested diagnostic group effects on the vigilance measures by using an age-adjusted subsample of *N* = 19 in each group in order to control for age effects. The age-matched groups differed statistically significantly in vigilance stability, and higher stability scores occurred in the depressive group, meaning a faster vigilance decline in the ADHD group compared to the depressive children. In the depressive group, we found statistical significant lower percentages of less alerted vigilance stage B23 (see Figure 2 and Table 4 for more details).

**Table 4:** EEG vigilance measures in all subsamples.

**Sample**		**Group (*N*)**	**Stability**	**Mean vigilance**	**0**	**A1**	**A2**	**A3**	**B1**	**B23**
All eligible	Mean (SD)	ADHD (76)	5.92 (1.44)	**3.63 (1.08)**	**5.90 (10.46)**	**55.84 (28.79)**	0.89 (2.59)	**1.04 (2.31)**	**5.40 (7.96)**	**30.93 (27.09)**
patients		DEP (94)	6.14 (1.64)	**4.21 (0.81)**	**8.64 (11.48)**	**64.32 (28.71)**	2.65 (7.72)	**1.48 (5.74)**	**9.54 (14.34)**	**13.37 (18.85)**
	*Z*; *p*; *r*		−0.99; 0.32; 0.08	**−3.77;** **<0.001; 0.29**	**−3.25;** **<0.001; 0.25**	**−2.08; 0.04; 0.16**	−1.01; 0.31; 0.08	**−2.01; 0.04; 0.15**	**−2.53; 0.01; 0.19**	**−5.54;** **<0.001; 0.43**
CBCL	Mean (SD)	ADHD (66)	6.00 (1.45)	**3.64 (1.06)**	**5.11 (9.26)**	57.32 (28.4)	0.87 (2.7)	1.04 (2.31)	**4.92 (7.13)**	**30.74 (26.52)**
		DEP (77)	6.09 (1.62)	**4.18 (0.82)**	**9.06 (11.71)**	62.6 (29.35)	2.93 (8.43)	1.75 (6.31)	**9.57 (13.55)**	**14.08 (19.72)**
	*Z*; *p*; *r*		−0.44; 0.66; 0.04	**−3.19;** **<0.001; 0.27**	**−3.56;** **<0.001; 0.3**	−1.26; 0.21; 0.1	−1.5; 0.13; 0.13	−1.5; 0.13; 0.13	**−2.59; 0.01; 0.22**	**−4.88;** **<0.001; 0.41**
TAP	Mean (SD)	ADHD (54)	5.81 (1.47)	**3.63 (1.04)**	6.35 (10.15)	**54.89 (28.86)**	1.08 (2.96)	1.36 (2.67)	6.03 (7.89)	**30.29 (26.29)**
		DEP (28)	6.25 (1.76)	**4.41 (0.60)**	7.94 (9.87)	**71.26 (26.60)**	1.62 (3.62)	0.96 (3.02)	9.51 (13.22)	**8.71 (11.76)**
	*Z*; *p*; *r*		−1.21; 0.23; 0.13	**−3.38;** **<0.001; 0.37**	−1.38; 0.17; 0.15	**−2.68; 0.01; 0.3**	−0.35; 0.73; 0.04	−1.67; 0.09; 0.18	−1.24; 0.21; 0.14	**−4.38; 0; 0.48**
Age-matched	Mean (SD)	ADHD (19)	5.53 (1.61)	4.17 (0.65)	10.08 (12.78)	62.22 (23.17)	2.27 (4.61)	1.38 (2.23)	7.90 (10.17)	**16.14 (14.23)**
		DEP (19)	6.58 (1.74)	4.31 (0.77)	8.62 (12.16)	67.96 (31.04)	1.04 (2.01)	1.27 (3.65)	10.04 (14.21)	**11.08 (17.13)**
	*Z*; *p*; *r*		**−1.981; 0.048; 0.32**	−1.299; 0.194; 0.21	−0.044; 0.965; 0.01	−1.445; 0.148; 0.23	−1.13; 0.258; 0.18	−0.79; 0.43; 0.13	−0.438; 0.661; 0.07	**−2.409; 0.016; 0.39**

*Mean and standard deviations (in parentheses) of all subsamples are shown. The statistical significant group differences are shown in bold. Less vigilant classification is existent in depressive patients in all subsamples, in particular lower percentage of B23 stage. Because the youngest patients are mainly patients with the diagnosis of ADHD and the eldest mainly are patients with diagnosis of depression, it is not possible to determine to what extent the group differences in the non-age-matched samples is due to age effects or to the diagnosis. Notably, covariate correlation analysis with age revealed differences in mean vigilance and B23 classification only in the whole sample, not for each diagnostic group separately. See Analysis of Covariates section. **Table 6** CBCL, Child Behavior Check List/6-18; TAP, Testing of Attentional Performance; r = Z/sqrt(N), Pearson's correlation coefficient as effect size measure for the Mann–Whitney U-test*.

**Table 5:** Correlation statistics of problematic behavior, attentional performance, and EEG vigilance.

		**Vigilance measure**			
	**Stability**	**Mean vigilance**	**A1**	**B23**	
**ADHD**	**CBCL**				
	Internalizing score	−0.17; 0.18	−0.04; 0.75	−0.06; 0.61	0.03; 0.81
	Externalizing score	−0.2; 0.1	−0.18; 0.15	−0.23; 0.06	0.21; 0.09
	Total score	**−0.289; 0.02**	−0.14; 0.26	−0.2; 0.11	0.15; 0.22
	Withdrawn/Depressed	−0.03; 0.83	0.09; 0.5	0.03; 0.78	−0.09; 0.47
	Somatic complaints	−0.16; 0.21	−0.16; 0.2	−0.12; 0.33	0.17; 0.19
	Anxious/Depressed	−0.11; 0.37	−0.07; 0.6	−0.08; 0.54	0.06; 0.61
	Social problems	**−0.257; 0.04**	−0.06; 0.63	−0.18; 0.14	0.05; 0.72
	Thought problems	**−0.285; 0.02**	−0.15; 0.24	−0.18; 0.16	0.13; 0.3
	Attention problems	−0.22; 0.08	−0.07; 0.59	−0.11; 0.4	0.07; 0.6
	Rule-breaking behavior	−0.18; 0.16	−0.14; 0.28	−0.18; 0.16	0.16; 0.21
	Aggressive behavior	−0.23; 0.06	−0.21; 0.1	−0.24; 0.05	0.24; 0.06
	**TAP**				
	Go error	0.04; 0.81	−0.02; 0.91	0.03; 0.85	0.03; 0.87
	Tonic alertness rt	−0.15; 0.31	0.07; 0.65	0; 0.98	−0.1; 0.48
**DEP**	**CBCL**				
	Internalizing score	−0.16; 0.16	0.01; 0.95	−0.04; 0.76	0.07; 0.53
	Externalizing score	0.04; 0.73	0.1; 0.41	0.18; 0.12	0.07; 0.52
	Total score	−0.12; 0.3	−0.02; 0.89	−0.01; 0.91	0.12; 0.31
	Withdrawn/Depressed	−0.09; 0.45	0.01; 0.96	0.03; 0.79	0.06; 0.61
	Somatic complaints	−0.01; 0.91	0.1; 0.38	0.05; 0.67	−0.1; 0.4
	Anxious/Depressed	−0.11; 0.33	0.07; 0.56	0.01; 0.96	0; 0.99
	Social problems	−0.03; 0.81	0.07; 0.54	0.17; 0.14	−0.13; 0.27
	Thought problems	−0.04; 0.73	−0.07; 0.58	0; 0.98	0.08; 0.5
	Attention problems	−0.09; 0.45	−0.01; 0.93	0; 1	0.01; 0.93
	Rule-breaking behavior	−0.07; 0.59	0.05; 0.66	0.06; 0.63	0.07; 0.58
	Aggressive behavior	0.07; 0.53	0.08; 0.47	0.2; 0.09	0.03; 0.8
	**TAP**				
	Go error	0; 0.99	−0.14; 0.49	−0.03; 0.88	0.09; 0.67
	Tonic alertness rt	0.17; 0.39	0.24; 0.25	0.28; 0.17	−0.3; 0.14

*Shown are the Spearman's correlation coefficient rho and the p-value. The statistically significant correlations are shown in bold. CBCL, Child Behavior Check List/6-18; TAP, Testing of Attentional Performance, psytest.de*.

### Analysis of Associations Between Vigilance, Behavioral Problems, and Attentional Performance

The correlation analysis revealed no statistically significant association of any vigilance measure with the behavioral problems, measured by CBCL. Additionally, we did not find an effect of EEG vigilance measures on the attentional performance, in particular no effects on the response time in the alertness task and no effect on the Go errors in the GFO/NoGo task.

### Analysis of Covariates

#### Time of EEG Acquisition

**Figure 3:**
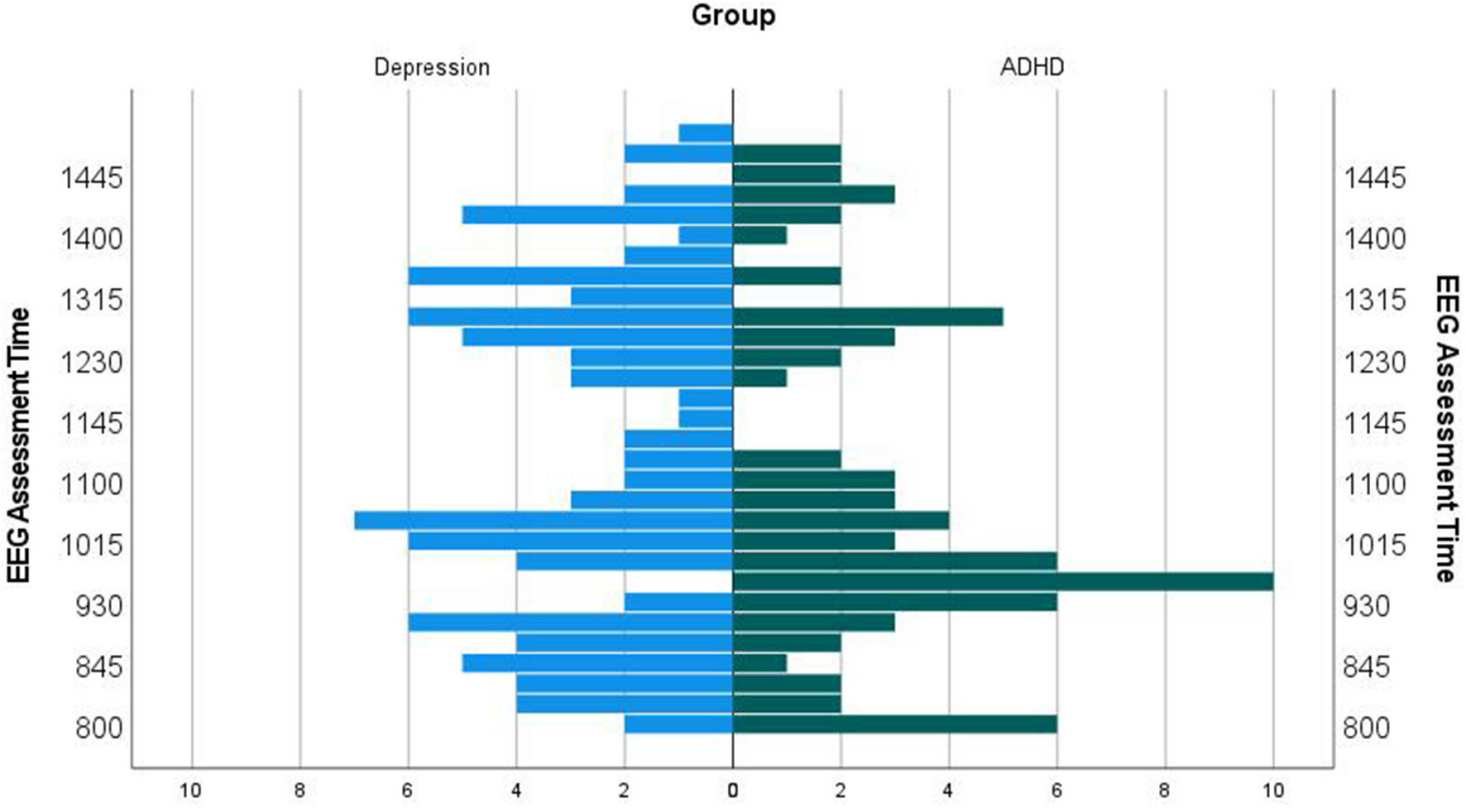
EEG assessment time. The time of the EEG assessment has no effect on the EEG vigilance measures.

The acquisition time distribution of all eligible patients is shown in Figure 3.

There was no statistically significant correlative association between time of EEG acquisition and EEG vigilance measures (Table 6). Additionally, the mean EEG assessment time was not different between the diagnostic groups in the age-matched sample: Mean/SD of assessment time, ADHD: 11:30 a.m./2.3 h, depression: 11:45 a.m./2.2 h, *T*_(36)_ = −0.39, *p* = 0.70, *d* = 2.27.

**Table 6:** Correlation statistics of age and assessment time and EEG vigilance.

**Sample**	**Value**	**Vigilance measure**			
		**Stability**	**Mean vigilance**	**A1**	**B23**
Both groups	Age	0.01; 0.88	**−0.312;** **<0.001**	0.14; 0.07	**−0.433;** **<0.001**
ADHD	Age	**−0.249; 0.03**	0.13; 0.25	−0.04; 0.73	−0.19; 0.1
	assessment time	−0.05; 0.65	0.1; 0.4	0.09; 0.46	−0.05; 0.69
DEP	Age	0.07; 0.51	0.18; 0.08	0.12; 0.25	−0.19; 0.06
	assessment time	−0.06; 0.57	0.08; 0.42	0.08; 0.45	0.03; 0.78

*Shown are the Spearman's correlation coefficient rho and the p-value: rho; p-value. The statistically significant correlations are shown in bold*.

#### Age

**Figure 4:**
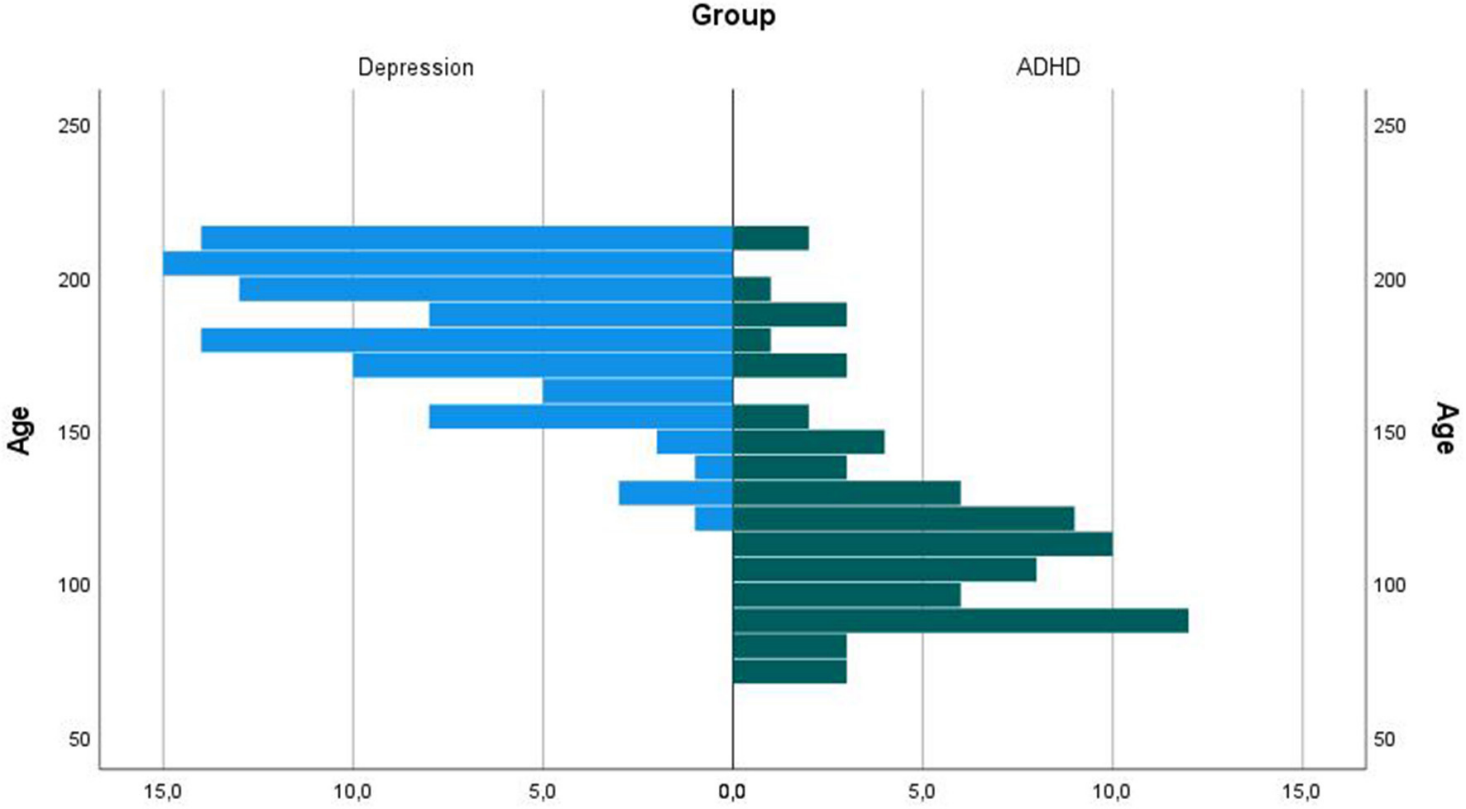
Age of participants. This is the age histogram of the all-eligible participant subsample, *N* = 76 (ADHD), *N* = 94 (Depression). As can be seen, the ages are differently distributed; the oldest participants are in the group of children with diagnosis of depression and the youngest participants are mainly in the group of children with ADHD diagnosis. In order to control for possible age effects on the EEG vigilance, we compared the vigilance measures between the diagnostic groups in an age-matched subsample of 19 participants in each group with a 2-month age difference.

The diagnostic groups of all eligible patients differed in age with a mean difference of 5.5 years, as can be seen in Figure 4 and Table 2.

**Figure 5:**
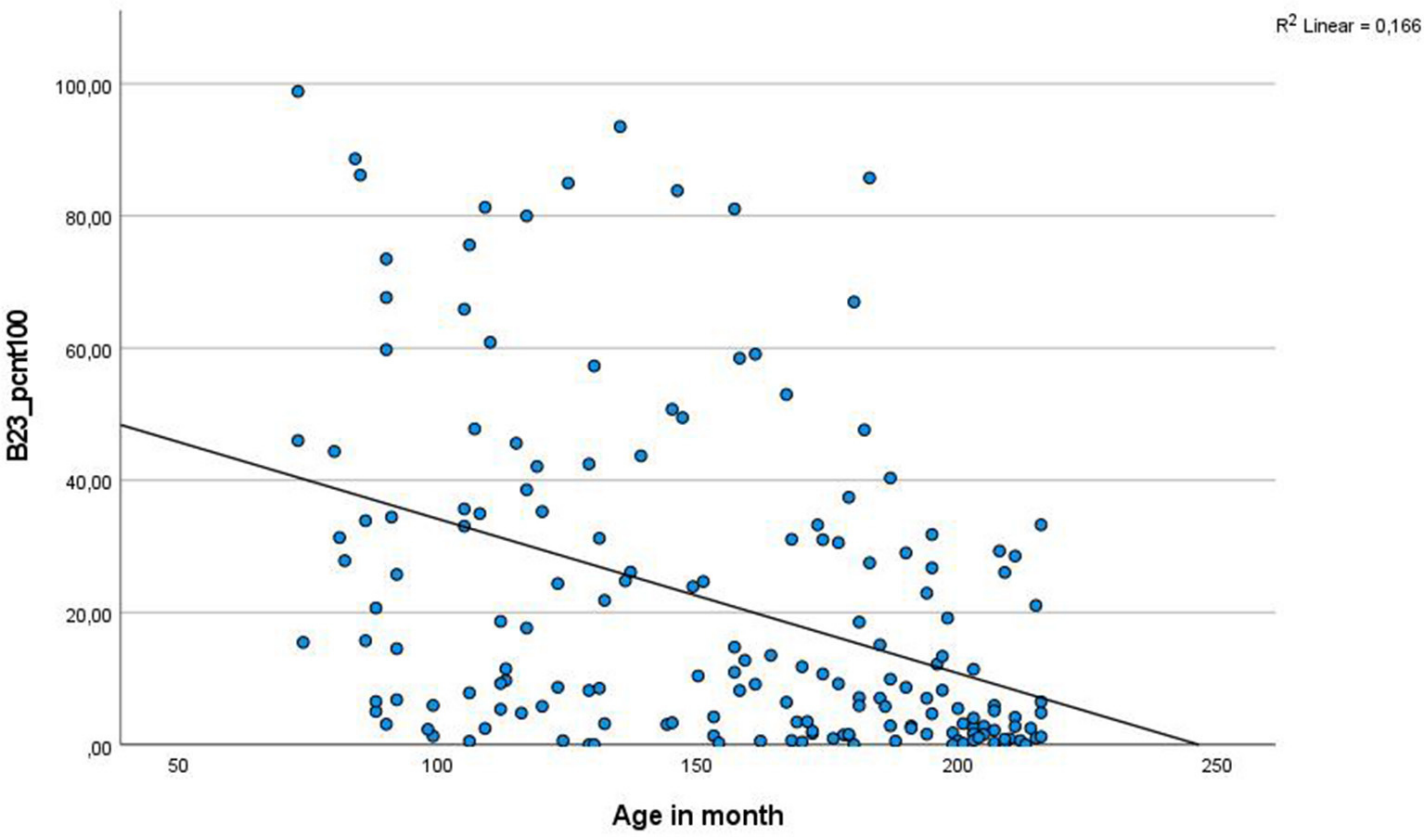
Correlation between age and EEG vigilance stage B2/3. Here, the scatter plot between age and vigilance classification of stage B23 (drowsiness) of all eligible patients can be seen. The age drowsiness association is mainly due to the difference between the youngest and the eldest patients, but who also differed in their diagnosis in our study. Therefore, it is not decidable here whether this effect is caused by the diagnosis, by age, or by a mixture of both. The B23 difference was significant in all tested subsamples (see Table 4).

The above-described age-dependent changes in EEG activity from childhood to adolescence, e.g., the increase and stabilization of posterior dominant rhythms until the age of 16 (30), also probably influence EEG vigilance measures. Therefore, as expected, we were facing age effects on EEG vigilance measures in this study. Age was negatively associated to vigilance stage; in particular, B23 stage occurrence decreased in children of higher age (Figure 5). Nevertheless, these age effects only occurred in the analysis of all participants combined, including the eldest and the youngest children for both diagnostic groups. In correlation analysis of vigilance and age relations for each diagnostic group separately, we did not find a significant correlation between age and vigilance level (see Table 6).

#### Medication

As described before, 30% of the patients received medication at time of EEG (see Table 7 for more details), and we analyzed any possible effects of this medication on the vigilance regulation and arousal level. We did not find any effects of the received medication on the EEG vigilance: There was no statistically significant difference in vigilance measures comparing medication yes or no with Mann–Whitney test and additionally there was no significant difference when we tested for each kind of received medication separately with Kruskal–Wallis test (see Table 8). However, the validity of this result is somehow limited because the obtained power was low. For comparing effects of received vs. no medication, given an alpha error of 0.05 and a power of 80% and the low sample size in our study (ADHD, no: 42/yes: 34; depression, no: 79, yes: 15), we could only detect medium effect sizes of about Cohen's *d* = 0.47 for the group of children with ADHD and *d* = 0.57 for the group of children with depression. In our study, we achieved an effect size *d* between 0.1 and 0.23. Because of the small sample size and the explorative character of this study, we did not include the medication as a covariate in the analysis.

**Table 7:** Received medication and related EEG vigilance.

		**No medication**	**Neuroleptics**	**Stimulants**
ADHD	*N*	42	11	23
	Stability	6.07; 1.24	5.82; 1.66	5.7; 1.69
	Mean vigilance	3.59; 1.02	3.42; 1.21	3.8; 1.13
	0	3.69; 5.81	6.99; 13.18	9.4; 14.44
	A1	58.06; 26.93	49.28; 35.83	54.94; 29.3
	A2	0.71; 2.44	0.64; 1.72	1.32; 3.19
	A3	1.02; 2.55	0.39; 0.6	1.39; 2.35
	B1	4.32; 5.71	7.6; 13.1	6.32; 8.47
	B23	32.19; 25.93	35.11; 30.5	26.62; 28.17
		**No medication**	**Neuroleptics**	**Antidepressants**
Depression	*N*	79	5	10
	Stability	6.19; 1.63	6.4; 1.67	5.6; 1.84
	Mean vigilance	4.19; 0.81	4.21; 1.12	4.38; 0.69
	0	8.78; 11.84	3.77; 4.2	9.97; 11.08
	A1	63.22; 28.3	70.74; 35.09	69.8; 30.98
	A2	3.1; 8.35	0.47; 0.84	0.12; 0.25
	A3	1.74; 6.23	0.17; 0.39	0.12; 0.26
	B1	9.21; 12.91	17.85; 32.34	7.94; 13.16
	B23	13.94; 19.35	6.98; 8.16	12.05; 19.23

*Shown are the mean and standard deviations (SD) of the vigilance measures, separated by semicolon: mean; SD. This is the subsample of all eligible patients N = 76 (ADHD), N = 94 (Depression). No antidepressive medication was given to patients with ADHD and no stimulants were given to the patients with depression*.

**Table 8:** No medication effects on vigilance.

		**Stability**	**Mean vigilance**	**0**	**A1**	**A2**	**A3**	**B1**	**B23**
**General use of medication (yes/no)**									
ADHD	*Z*; *p*; *r*	−0.71; 0.477; 0.05	−0.845; 0.398; 0.06	−0.543; 0.587; 0.04	−1.432; 0.152; 0.11	−0.628; 0.53; 0.05	−0.57; 0.568; 0.04	−0.763; 0.446; 0.06	−0.71; 0.477; 0.05
Depression	*Z*; *p*; *r*	−0.4; 0.689; 0.03	−0.918; 0.359; 0.07	−0.353; 0.724; 0.03	−0.735; 0.462; 0.06	−1.076; 0.282; 0.08	−0.871; 0.384; 0.07	−0.155; 0.877; 0.01	−0.4; 0.689; 0.03
**Use of specific medication**									
ADHD	*H*; *p*; η^2^	1.323; 0.516; 0.01	1.428; 0.49; 0.01	0.522; 0.77; 0.02	3.407; 0.182; 0.02	2.251; 0.324; 0	0.331; 0.847; 0.02	1.359; 0.507; 0.01	1.323; 0.516; 0.01
Depression	*H*; *p*; η^2^	1.41; 0.5; 0.01	1.64; 0.44; 0	3.88; 0.14; 0.02	3.3; 0.19; 0.01	0.92; 0.63; 0.01	0.81; 0.67; 0.01	1.41; 0.5; 0.01	1.64; 0.44; 0

*Here are shown the statistic results of the analysis of medication effect on vigilance measures. Analysis of general use (yes/no) of medication was performed with Mann–Whitney U-test. The use of specific medication was analyzed with Kruskal–Wallis test. Statistical parameter Z for Mann–Whitney, H for Kruskal–Wallis. Effect size parameter: r, Pearson's regression coefficient = Z/$\sqrt{n}$ and η^2^= (H – k + 1)/(n – k); n, number of subjects; k, number of groups (see Table 7)*.

## Discussion

In this study, EEG measures acquired from a convenience sample of in- and outpatients of the childhood psychiatry department diagnosed with ADHD or depression were analyzed for vigilance effects with VIGALL 2.1. As has already been shown for adult patients with ADHD and depression, we found lowered vigilance level and a faster vigilance decline in a sample of children and adolescents with ADHD compared to patients with depressive symptomatology.

This study confirms the several findings of divergent arousal regulation in adult samples of ADHD and depression in a sample of children and adolescents. To our knowledge, there has been no study measuring EEG vigilance in depressive children and only one study comparing ADHD children with healthy controls (18). Therefore, this study is the first one, investigating the arousal regulation model for ADHD and affective disorders (1) in children directly by comparing children and adolescents with ADHD and depression. Moreover, our data suggest that even the standard procedure of the clinical routine EEG (resting state), which is not specialized to vigilance assessment, can be used to differentiate brain arousal states between patients with ADHD and depression. Therefore, this study should encourage further EEG research analyzing brain arousal patterns in additional types of mental disorders, using recordings from larger databases and routine, clinical EEGs, including retrospectively.

We analyzed the possible effect of moderator variables such as received medication, time of EEG acquisition, and age of the participants and allowed the relevance of the covariates to determine our further statistical approach. We found no statistically significant difference in the vigilance measures between the categories of received medication. Because the sample size was small, the inference of our covariate analyses is limited. A proposed analysis is equivalence tests, performed on larger samples in future studies, e.g., by comparing the confidence interval of observed effect sizes with the smallest effect size of interest (SESOI) (31). In our study, the SESOI is increased by the effect size we could reliably detect, but because of the exploratory design, we furthermore analyzed only EEG data in accordance to the constraints made by the VIGALL toolbox, regardless of received medication. Nevertheless, because we are aware of no prior report of medication effect sizes in relation to EEG vigilance measures in children, we believe that the covariates analysis will be meaningful for sample size estimation when planning future studies.

This study was conducted on clinical, routine, EEG data acquired on psychiatric patients, and the data were affected by some covariates that possibly have an impact on vigilance measures. Because of this, there are some limitations that need to be kept in mind. Firstly, there are established age-related changes in EEG measures, related to brain development. Age-related EEG changes include a decrease of absolute spectral EEG power at all frequencies at higher ages, possibly as a result of synaptic pruning during maturation (32). Relative spectral EEG power is decreasing with brain development at Delta and Theta, but increasing for higher frequencies (Alpha, Beta, and Gamma). Changes in vigilance, however, are not triggered by changes in topography; the maps oscillating at lower frequencies in lower ages have the same topography but higher frequencies in older groups (33). Another important change during brain development is the individual Alpha peak frequency (iAPF), which has been found to increase to adult values around the age of 11, although further increases may still be present until the age of 15 (30, 34). Recently, in a comprehensive review (35), the developmental change in the resting-state EEG was assigned to different developmental periods, from infancy, over adolescence to adulthood and includes also findings on functional connectivity and networks.

The toolbox VIGALL assumes a stable iAPF in the range from 8.5 Hz up to 12.5 Hz, which was a filter criterion for data inclusion in this study and therefore age-related changes in iAPF should not limit the vigilance classification. In this study, we have seen a significant correlation between age and vigilance level measures, but this correlative effect did not exist in correlation analysis for each diagnostic group separately. Due to the particular characteristics of our samples, it was not possible to separate and explore age and diagnostic group in the correlation analyses. All of the eldest adolescent patients and only a few of the youngest patients had the diagnosis of depression. Therefore, it was not possible to know the extent to which the statistical age effect on EEG vigilance was caused by altered brain arousal associated with the psychiatric diagnosis or caused by normal differences of developmental brain states, e.g., the increased relative spectral power in the Theta frequency in younger brains. The size of our consecutive sample, however, allowed us to draw a sub-sample matched for age in order to compare the groups. The groups differed from each other in vigilance measures and so this study suggests that the different vigilance regulation types that have been found in the adult EEG (1) are probably also existent in children and adolescents.

Nevertheless, beside the group differences of vigilance level and decline, there was no association between the vigilance parameter and the problematic behavior of the children, measured by the parents' rating via the child behavior checklist. In particular, in the present study, there was no association between vigilance measures and attentional problems, withdrawn-depressive, or anxious-depressive behavior.

In contrast, such associations between symptomatology and brain arousal have been found in adult studies. In a first study using EEG vigilance measures in non-medicated depressive patients, results revealed a moderate association between clinicians' ratings of depression severity (HDRS-17) and vigilance substages A1, B1, and B2/3, but no significant correlation between vigilance measures and self-ratings of depression severity (BDI) (9). Recently, higher arousal level and a slower arousal decline corresponded to higher severity of depressive symptoms measured by BDI in a sample of SSRI-medicated depressive patients (36). In another study by this research group, a sample of adult ADHD patients were divided into a stable and an unstable group, regarding their arousal decline during the EEG measurement. The participants in the slower arousal declining group reported more depressive symptoms than the unstable group, but no association was found to ADHD symptomatology (37). In a study on adult individuals with diagnosis of ADHD, a multiple regression analysis indicated that retrospectively assessed severity of childhood ADHD symptoms was associated to arousal regulation (15). The lack of association between vigilance parameter and symptomatology in the present study could not be fully explained with the present data. Routine EEG procedure may be sensitive to find vigilance differences between clinical groups, but the variance in the vigilance time series of monitored EEG acquisitions could be too low to analyze association with task performance or symptom severity. It is still an open question whether a possible association exists, and if so, possibly it could be revealed using EEG assessments of longer duration without monitoring the alertness of the participants during EEG acquisition and therefore increasing variability in the EEG vigilance measures. Additionally, for a detailed analysis of developmental changes of brain arousal, it would be preferable to include a larger sample, with a smaller age range.

Nevertheless, it should be emphasized that the further topography and frequency-related analysis of clinical routine EEG data with tools like VIGALL for assessment of vigilance regulation could provide an additional diagnostic value. Related to brain arousal, Hegerl and Ulke (38) stated that clinicians have to verify whether patients' motivational problems with fatigue have their cause in hypoarousal with apathy, sleepiness, and lack of drive or in hyperaroused brain states with exhaustion, inhibition of drive, and ambivalence. The evaluation of vigilance regulation profiles from routine EEG could practically support the distinction between these different forms of fatigue with implications for further treatment.

The findings of the different vigilance level and decline between children with diagnosis of ADHD and children with a diagnosis of most prominent depressive symptoms are firstly limited by the small number of only 19 age-matched participants in each group. Secondly, the effects of medication could possibly alter the vigilance regulation, despite the fact that we did not find any medication effects on the EEG arousal. With these limitations, this study has an explorative character and should be repeated on a larger sample, free of any medication. Nevertheless, because we have controlled for all covariates, the findings of this study could be treated as first evidence that similar differences in vigilance regulation processes exist in children with ADHD and depression as has been shown for adult samples.

## Data Availability Statement

The data that support the findings presented in the study are available from the corresponding author/s upon reasonable request.

## Ethics Statement

The studies involving human participants were reviewed and approved by Ethics Committee of Rostock University Medical Center, Rostock, Germany. Written informed consent from the participants' legal guardian/next of kin was not required to participate in this study in accordance with the national legislation and the institutional requirements.

## Author Contributions

CB designed and conceptualized the study, analyzed the data, and drafted the manuscript for intellectual content. AD helped in conceptualizing the study. FP acquired the data. KW acquired and interpreted the data, and helped in conceptualizing the study. JB interpreted the data and revised the manuscript for intellectual content. MK revised the manuscript for intellectual content. OR helped in study design and conceptualizing the study, and revised the manuscript for intellectual content. IM helped conceptualizing and analyzing the data, and drafted the diagnostic procedure description for the Methods section. All authors contributed to the article and approved the submitted version.

## Conflict of Interest

The authors declare that the research was conducted in the absence of any commercial or financial relationships that could be construed as a potential conflict of interest.

## Publisher's Note

All claims expressed in this article are solely those of the authors and do not necessarily represent those of their affiliated organizations, or those of the publisher, the editors and the reviewers. Any product that may be evaluated in this article, or claim that may be made by its manufacturer, is not guaranteed or endorsed by the publisher.
